# High Seebeck Coefficient of Porous Silicon: Study of the Porosity Dependence

**DOI:** 10.1186/s11671-016-1411-z

**Published:** 2016-04-14

**Authors:** Katerina Valalaki, Philippe Benech, Androula Galiouna Nassiopoulou

**Affiliations:** Institute of Nanoscience and Nanotechnology (INN), NCSR Demokritos, Terma Patriarchou Grigoriou and Neapoleos, Athens, 153 10 Greece; IMEP-LAHC, Grenoble Institute of Technology - Minatec, Parvis Louis Neel 3, Grenoble, 38016 France

**Keywords:** Porous Si, Seebeck coefficient, Thermoelectrics, Low-dimensional semiconductors, 60, 68.65k, 65.80g

## Abstract

In-plane Seebeck coefficient of porous Si free-standing membranes of different porosities was accurately measured at room temperature. Quasi-steady-state differential Seebeck coefficient method was used for the measurements. A detailed description of our home-built setup is presented. The Seebeck coefficient was proved to increase with increasing porosity up to a maximum of ~1 mV/K for the ~50 % porosity membrane, which is more than a threefold increase compared to the starting highly doped bulk c-Si substrate. By further increasing porosity and after a maximum is reached, the Seebeck coefficient sharply decreases and stabilizes at ~600 μV/K. The possible mechanisms that determine this behaviour are discussed, supported by structural characterization and photoluminescence measurements. The decrease in nanostructure size and increase in carrier depletion with increasing porosity, together with the complex structure and morphology of porous Si, are at the origin of complex energy filtering and phonon drag effects. All the above contribute to the observed anomalous behaviour of thermopower as a function of porosity and will be discussed.

## Background

Silicon is the basic material of semiconductor electronics, and therefore, there is huge infrastructure and know-how available for its production and processing. It is thus highly advantageous to combine silicon with other devices towards both lowering fabrication cost and more importantly adding more functionality to Si by combining logic and memories with other functions (system-on-chip (SoC)). Devices to be integrated on Si include thermoelectrics (TE), for which a lot of research is carried out towards increasing the efficiency of Si-based and Si-compatible thermoelectric materials. Solid-state thermoelectric modules converting heat to electricity and vice versa are of increasing demand for use in power generation [1] and cooling or refrigeration [2–5], respectively. Si itself is a poor thermoelectric material since it conducts heat very well when it is in bulk crystalline form. A global trend in the development of thermoelectric materials is towards nanostructured and composite materials, with the overall objective to achieve at the same time high Seebeck coefficient and low thermal conductivity. Concerning Si, researchers showed that by nanostructuration, the Seebeck coefficient is enhanced and the thermal conductivity is reduced; thus, the figure of merit ZT (ZT = *σS*^2^*T*/(*k*_e_ + *k*_l_)), where *σ* is the electrical conductivity, *S* the Seebeck coefficient, *T* the absolute temperature, and *k*_e_ and *k*_l_ the electronic and lattice components of thermal conductivity, respectively, is increased.

The most promising form of nanopatterned Si studied so far is the so-called holey silicon [6], in which vertical pores were created with the aim to reduce thermal conductivity without compromising the electrical conductivity. A figure of merit ZT ~ 0.4 at room temperature was demonstrated, which is much higher than that of highly doped bulk crystalline Si (~0.01). Apart from holey silicon, other forms of nanostructured Si are currently studied for application in thermoelectrics. One such example is Si nanowires, which were demonstrated to exhibit high ZT values [7, 8]. Theoretical works also exist, showing enhanced thermoelectric properties of nanoengineered porous Si [9, 10]. However, instead of using nanoengineered Si fabricated with complicated patterning techniques [11], a more massive and low-cost technique to produce porous Si (PSi) is electrochemistry. By electrochemical etching of bulk crystalline silicon, PSi layers of different structure and morphology can be fabricated on bulk Si. Pores can be either randomly oriented in a sponge-like form or vertically oriented on the bulk substrate, with pore diameter and shape dependent on the electrochemical conditions used and the type and resistivity of the starting Si wafer material. Pores can be also filled with different materials, giving thus possibilities of further tailoring material properties [12–15].

Although electrochemically etched PSi seems to be a promising material for use as a thermoelectric material, only few experimental works were devoted to this subject [16–18]. In the corresponding papers, high-porosity samples (porosity above 50 %) were in general used, which are not the best choice. Indeed, by increasing the porosity, the thermal conductivity is considerably decreased [19–21]; however, the electrical conductivity is also highly decreased. The study of lower porosities seems to be a better strategy.

In this paper, we systematically studied the Seebeck coefficient (*S*) of PSi with porosities ranging from 40 to 85 %. Our objective is to continue with lower porosities and the characterization of all their thermoelectric parameters. For our measurements, we used free-standing PSi membranes and we systematically assessed our experimental setup concerning its design, contact geometry and data acquisition in order to guarantee accurate and reliable measurements. This was a necessary step since there are conflicting results in the literature resulting from both differences in measurement accuracy and materials’ structure and morphology.

## Methods

**Fig. 1:**
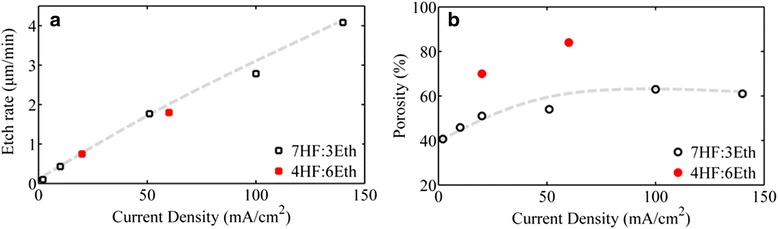
Etch rate and porosity versus the anodization current density for two different electrolytes. The etch rate (**a**) and the porosity (**b**) as a function of the current density used in electrochemistry for two different electrolyte compositions are depicted. *Dashed lines* are guides to the eye

PSi free-standing membranes were fabricated at room temperature by electrochemical etching of highly doped p-type c-Si wafer with resistivity of 1–5 mΩ.cm in a single-tank Teflon cell in the dark. Porosification was made on predefined areas of the Si wafer through a masking layer composed of a bilayer of polycrystalline Si/SiO_2_ [22]. A two-step electrochemical process was used for PSi membrane formation and detachment from the substrate. In the first step, the current densities used were below the critical value *J*_ps_ [23] for electropolishing. More specifically, the current densities between 2 and 100 mA/cm^2^ were used, resulting in PSi layers with different porosities. In the second step, the current density was increased above *J*_ps_ (~500 mA/cm^2^) in order to detach the membranes from the Si substrate. In both steps, the electrolyte used was a mixture of hydrofluoric acid (HF) and ethanol in two different volume concentrations, namely 7HF:3ethanol or 4HF:6ethanol. The electrolyte composition was changed because it is difficult to achieve low and high porosities with the same electrolyte. Figure 1a, b shows, respectively, the etch rate and porosity as a function of the current density used for the two different electrolytes. For the case of the 7HF:3ethanol electrolyte, porosity variation with the current density tends to saturate for the current densities above ~50 mA/cm^2^. It is thus necessary to change the electrolyte in order to achieve higher porosities at an acceptable etch rate.

Using the above electrochemical conditions, we fabricated free-standing PSi membranes with porosities between 40 and 84 %. For the porosity determination, the three-weight measurement method was used. Using this method, the porosity *P* is calculated by [24]

$$ P = \frac{m_1-{m}_2}{m_1-{m}_3}, $$

where *m*_1_ is the initial mass of the sample, *m*_2_ the mass after anodization and *m*_3_ the mass of the sample after removing the formed PSi layer.

The experimental parameters for the fabrication of the PSi membranes used in this work are summarized in Table 1.

**Table 1:** Electrochemical conditions used for PSi membrane formation

PSi	Electrolyte	Current density (mA/cm^2^)	Thickness (μm)	Anodization time (min)	Porosity (%)
1	3ethanol:7HF	2	64	495	41 ± 3
2	3ethanol:7HF	10	120	240	46 ± 2
3	3ethanol:7HF	20	100	128	51 ± 1
4	3ethanol:7HF	51	100	52	54 ± 1
5	3ethanol:7HF	100	100	36	63 ± 2
6	6ethanol:4HF	20	100	133	70 ± 4
7	6ethanol:4HF	60	100	55	84 ± 3

**Fig. 2:**
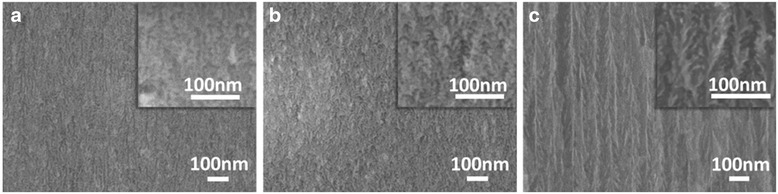
Cross-sectional SEM images of PSi membranes with three different porosities. Porous Si membranes with porosities 40, 46 and 70 % are depicted in **a**, **b** and **c**, respectively. The same images are depicted at higher magnification in the corresponding *insets*

Figure 2 shows examples of cross-sectional scanning electron microscopy (SEM) images of the fabricated PSi membranes. The insets show the same images at higher magnification.

**Fig. 3:**
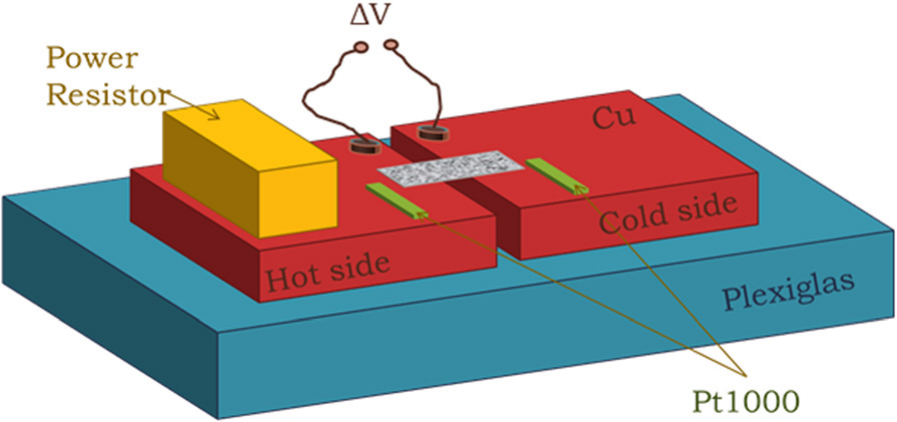
3D schematic representation of the home-built setup used for Seebeck coefficient measurements

A home-built setup was used for differential quasi-steady-state two-probe Seebeck coefficient measurements [25], which is schematically shown in Fig. 3. A plexiglas platform is used, on which two thick Cu blocks are fixed. The distance between them is 2 mm. One of these blocks serves as a “hot” contact and the other as a “cold” contact. The free-standing PSi membranes are fixed between the two blocks in the form of a bridge, attached to each block with silver paste. Good electrical contacts were thus achieved. A power resistor is placed on one of the Cu blocks and is used as a heater.

The experimental setup used for Seebeck coefficient measurements is shown in Fig. 3 in 3D representation. The two Cu blocks fixed on the plexiglas are shown, on which the power resistor and the two temperature-sensing resistors are fixed. There are also two wires fixed on each Cu block near the gap, which are used to measure the voltage difference between the “hot” and “cold” contacts of the membrane. One Cu block serves as the “hot” contact (heated by the power resistor) and the other as the “cold” contact.

A temperature difference (Δ*Τ*) is created between the two ends of the membrane by applying a dc current pulse of 1-min duration on the heater situated at the “hot” contact. By varying the amplitude of the current pulse, different heating rates can be achieved. The temperature is monitored for a total time of 30 min. Pt1000 resistance temperature detectors (RTDs) of class A are used for the accurate temperature measurement on each copper plate. RTDs are in general more accurate, precise and stable than thermocouples [26] frequently used for sensing temperature in Seebeck coefficient measurement setups. The two RTDs are fixed on each copper plate with a double-sided polyimide tape, which is thermally conductive and electrically insulating. Two Keithley 2400 source meters are used to determine the resistance of each RTD during measurement, and a Keithley 2000 digital multimeter is used to measure the resulting thermovoltage. All the instruments are connected to a PC using General Purpose Interface Board (GPIB) bus trigger. Data acquisition is performed by sequential data recording. Three experimental parameters are recorded, namely the resistance of each RTD at the cold and hot sides of the setup and the corresponding thermovoltage. The total time for recording all three parameters is 145.3 ms. Even though the total time for each complete set of measurements (*T*_hot_, *T*_cold_ and Δ*V*) is sufficiently short, additional care was taken in order to eliminate any possible error due to the different time at which each value is recorded, by introducing interpolated data [27]. In order to eliminate the effect of voltage offsets, the *S* values are extracted from the slope of the Δ*V* versus Δ*Τ* curve for Δ*Τ* below 2 K. The low Δ*Τ* assures no change in the *S* values, which could occur because of the increase in the temperature of the sample. The power resistor was switched off during Δ*V* and Δ*Τ* measurements in order to avoid any spurious electrical signal that could affect this measurement [25, 27].

In order to assess the measurement setup and the accuracy of our measurements, we first measured the Seebeck coefficient of the p-type silicon substrate with resistivity of 1–5 mΩ.cm (carrier concentration ~5 · 10^19^ atoms/cm^3^), from which our membranes were fabricated. A value of 299.3 μV/K was found, which is in agreement with values in the literature.

Photoluminescence (PL) measurements were also performed on our samples, and the obtained results were combined with structural measurements using cross-sectional scanning and transmission electron microscopy (SEM, TEM). For PL measurements, the Ar laser line at *λ* = 457.8 nm (2.7 eV) was used.

**Fig. 4:**
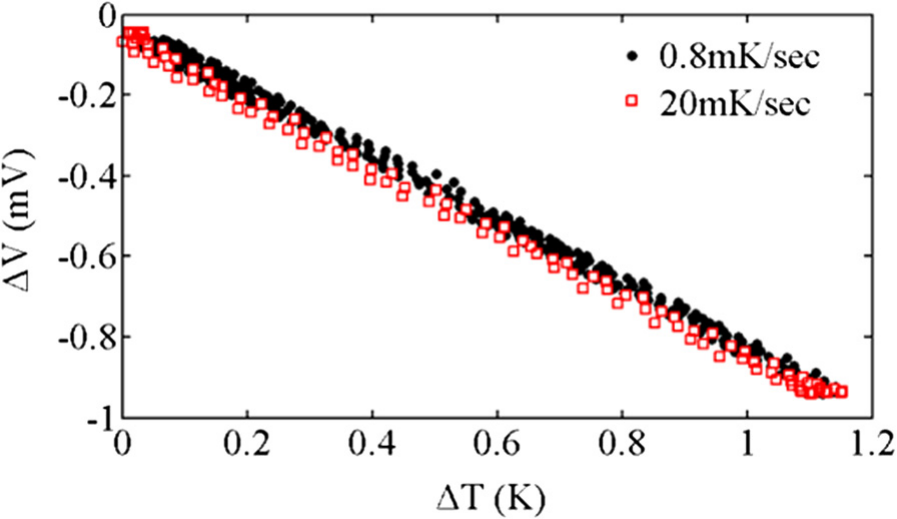
Diagnostic test measurement for hysteretic behaviour. Thermovoltage versus temperature difference for two heating rates, namely 20 mK/s (*red open squares*) and 0.8 mK/s (*black dots*). The *two curves* coincide, which is an indicator of good thermal contacts and the absence of hysteresis effect

We first tested the linearity and absence of hysteresis effect in the Δ*V* versus Δ*Τ* measurements. In this respect, we used two different heating rates, namely 20 and 0.8 mK/s and we performed measurements on a 40 % porosity sample, both by increasing and decreasing Δ*T*. The results are depicted in Fig. 4.

From Fig. 4, we deduce that the two different curves nicely coincide and there is no hysteresis effect between measurements obtained by increasing or decreasing Δ*Τ*. By using a linear fit to the two different sets of measurements, the *S* values of 804.7 μV/K for the 20 mK/s heating rate and 791.2 μV/K for the 0.8 mK/s heating rate were obtained. The dispersion between the two values is only ~1.7 %. This is an indicator that measurements with our system are accurate and do not exhibit any thermal or electrical contact problems.

**Fig. 5:**
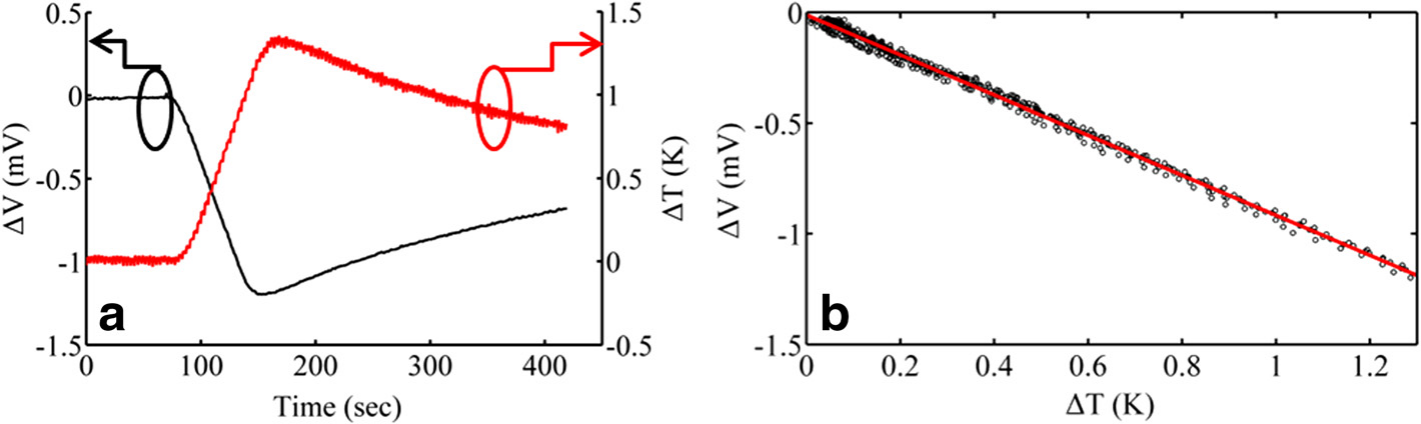
Example of experimental data for the 46 % porosity sample. In **a**, the thermovoltage and the temperature difference versus time are depicted for the 46 % porosity sample. In **b**, the Δ*V* versus Δ*T* curve is plotted, from which *S* is determined by a linear fit to the data

An example of Δ*V* and Δ*Τ* versus time data, obtained with a 46 % porosity sample, is given in Fig. 5a. In Fig. 5b, the Δ*V* versus Δ*Τ* curve is plotted, from which *S* is extracted using a linear fit to the data (see Fig. 5b) (red line), from which the value of *S* = 916.9 μV/K is obtained.

## Results and Discussion

Table 2 summarizes the *S* values obtained for seven different porosity samples.

**Table 2:** Porosity dependence of PSi Seebeck coefficient

Porosity (%)	40	46	51	55	63	70	84
S (μV/Κ)	781	917	1014	903	636	584	662

The measured *S* values of all PSi samples are positive, suggesting that electrical transport in the material is governed by hole transport, as expected by considering that the starting Si wafer was p-type. Holes are due to boron impurities in the “mother” Si wafer. This result is controversial to that reported by Mathur et al. [16], who reported negative values of *S* for PSi layers, formed also by anodization of p-type Si, as in the present work. On the other hand, our results agree in this respect with those reported by Yamamoto et al. [17] for PSi layers formed on p-type c-Si wafer with resistivity of 5 mΩ.cm.

**Fig. 6:**
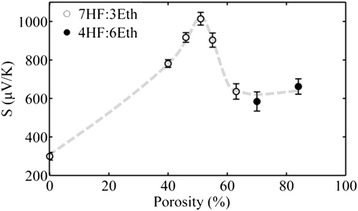
Porosity dependence of the Seebeck coefficient of PSi membranes. The *open circles* correspond to the electrolyte with 70 % HF concentration and the full ones with 40 % HF concentration as it is indicated in the legend. *Dashed line* is a guide to the eye

The Seebeck coefficient as a function of porosity for all samples measured is plotted in Fig. 6. The measured *S* value of crystalline Si (zero porosity) is also plotted for comparison. As stated above, unfortunately, we were obliged to change the HF:ethanol ratio of the electrolyte for the higher porosity samples. The exact conditions of the current density, electrolyte composition, anodization time and membrane thickness for all samples used in this work are summarized in Table 1.

From Fig. 6, we deduce that by increasing porosity, *S* increases up to a certain porosity value (maximum at ~50 % porosity), while for higher porosities, *S* decreases and stabilizes at around ~600 μV/K. The Seebeck coefficient of PSi is in all cases much higher than that of the corresponding bulk crystalline Si, used as the starting material. A similar trend of the Seebeck coefficient versus porosity was reported by Yamamoto et al. [17] using also PSi formed on highly doped p-type crystalline Si. However, the electrochemical solution used was slightly different (composition 1HF:1ethanol for all measurements); one should thus expect a slightly different material structure. They obtained an increase in *S* with porosity up to a certain value and then a sharp decrease; the observed maximum was however at a different porosity compared to our results. In addition, the decrease in *S* with porosity was more monotonous than in our case, where we see that *S* stabilizes at ~600 μV/K for porosities above ~60 % (see Fig. 6). In order to explain our results, we have to consider the structure and morphology of the PSi samples as a function of porosity.

**Fig. 7:**
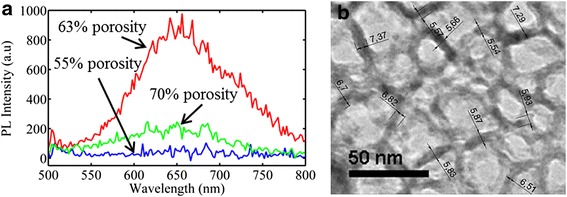
**a** PL spectra of porous Si membranes with different porosities. The sample with the highest porosity (*green line*) was fabricated using different electrolyte compositions (40 % HF concentration). **b** TEM image of the 70 % porosity sample. From the TEM image, the average pore wall thickness was calculated to be ~6 nm

PSi has a very complex material structure and morphology. It is composed of interconnected Si nanowires and nanocrystals surrounded by a shell native oxide and separated by voids (pores). By increasing porosity, the complexity of the material increases. The ratio between nanowires, nanocrystals and pores composing the material changes with porosity. This ratio does not depend only on porosity but also on the electrolyte used and the type and resistivity of the starting Si wafer. Multiple distinct phenomena are responsible for the porosity dependence of S. Apart from the increase of electrical resistivity with porosity due to carrier depletion, the interplay between energy-filtering effect, phonon drag and boundary scattering determines the *S* dependence on porosity. In order to better characterize the structure and morphology of our samples, photoluminescence measurements and TEM imaging were used. Figure 7a depicts the PL spectra of three PSi membranes with different porosities, while Fig. 7b shows a representative TEM image of the 70 % porosity sample. From Fig. 7a, we see that membranes with 55 % porosity do not emit any light. On the contrary, the 63 and 70 % porosity membranes emit red light, with the 63 % porosity sample being more efficient than the 70 % porosity sample. For higher porosities (results not shown here), PL was much more efficient. The above results shed some light concerning the structure and morphology of the samples.

Indeed, PL from PSi at room temperature starts to be efficient when the material comprises nanocrystals with mean crystallite size below ~5 nm, that is below the dimension of free exciton (~4.9 nm) of bulk crystalline Si. From the PL spectra of Fig. 7a, it is obvious that below 55 % porosity, our PSi membranes do not show any photoluminescence because, as deduced from the SEM image of Fig. 2b, the nanostructure size of the PSi skeleton is quite large (~20 nm). By increasing porosity, room temperature PL starts to appear. A strong peak at 650 nm is observed for the 63 % porosity sample, suggesting the existence of nanocrystals with sizes below ~5 nm within the Si skeleton. Strong quantum confinement starts to occur, leading to the appearance of photoluminescence. On the other hand, in the 70 % porosity sample, the mean size of nanostructures therein is higher, as it was confirmed both from the colour of that sample (blackish) and its TEM image depicted in Fig. 7b. From this image, we deduce that the mean distance of the Si walls of the PSi structure is ~6 nm, which explains the almost absence of PL from this sample. The weak PL peak observed is attributed to the fact that the percentage of tiny nanocrystals with size below ~5 nm is smaller than in the case of the 63 % porosity sample, which shows much more intense red PL (peak at 650 nm (1.9 eV)). The above anomalous variation of structure with porosity is not surprising in the case of our samples, resulting from electrochemical etching of highly doped p^+^ Si. Contrary to the sponge-like structure of the p-type-etched Si, in this case, the material structure is anisotropic, composed of vertical pores and wires with a complicated branching on their surface. We observed that the ratio of nanocrystals and nanowires composing their skeleton does not change monotonically with porosity. However, we have to note here that the influence of nanostructure size on *S* is not the same as on PL. Indeed, in Si nanocrystals of 5 nm, there are large differences in PL by changing the size from 1 to 2 nm, which is not the case for S.

**Fig. 8:**
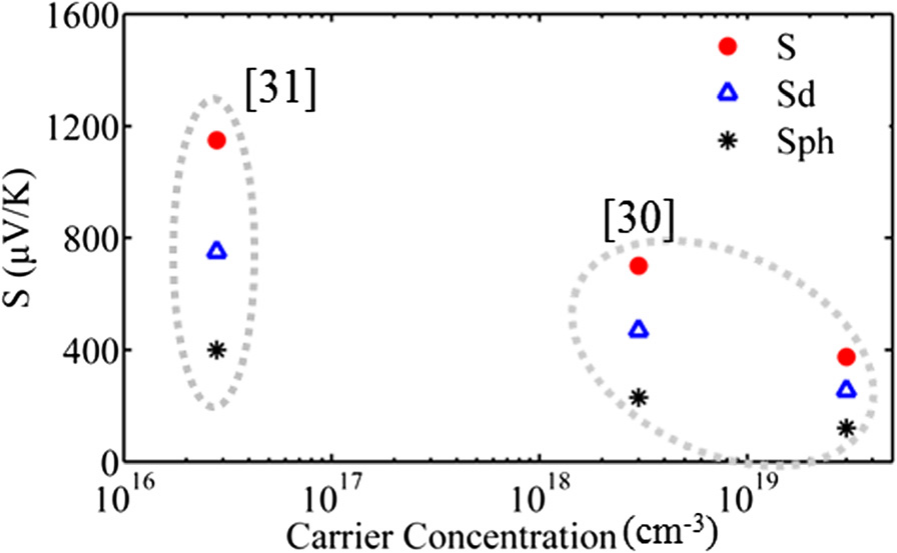
Total Seebeck coefficient and contributions of *S*_d_ and *S*_ph_ in bulk c-Si at different carrier concentrations. *S*, *S*_d_ and *S*_ph_ were deduced from two different references in the literature [30, 31]

The difference in structure and morphology of our different porosity samples are at the origin of the observed complicated *S* variation with porosity. In general, the Seebeck effect arises from the diffusion of charge carriers along a temperature gradient and it is enhanced by the drag imposed to carriers from the accompanying diffusion of phonons. The thermoelectric power *S* is thus expressed as the sum of a diffusion part *S*_d_ and a phonon drag part *S*_ph_. The diffusion part results from the spatial variation of the occupation probability of the carriers, caused by the temperature gradient along the sample, while the phonon drag part is due to momentum transfer from the phonon system to the electron one by electron-phonon scattering. In low-dimensional semiconductors and porous media, different effects affect the above two factors. The low dimensionality in a porous material introduces quantum confinement and strong scattering effects, affecting carrier and phonon transport. Most authors in the literature attribute the large *S* in porous materials to the strong scattering in material boundaries, which is accompanied by energy filtering effects [17, 28, 29], resulting in *S*_d_ enhancement. Energy filtering results from the more efficient scattering of low-energy carriers compared to high-energy carriers. Consequently, the Seebeck coefficient, which measures an average energy of electrons contributing to electrical conductivity, is enhanced. The coupling between electrons and phonons resulting in phonon drag has to be also considered. There are limited data in the literature concerning phonon drag in low-dimensional systems. In bulk semiconductors at room temperature, the phonon drag effect is in general considered to be important in the case of the undoped or lightly doped material and smaller in the heavily doped material. Figure 8 shows some representative data from the literature [30, 31] relating *S*, *S*_d_ and *S*_ph_ with doping concentration in the case of bulk Si. A large dependence of *S* on doping is observed.

In order to understand the variation of the Seebeck coefficient versus porosity in the case of our samples, we have to take into account the low dimensionality of our material. PSi is a complex system, composed of a skeleton of interconnected nanowires and nanocrystals, separated by voids. These nanostructures are depleted from carriers, with carrier concentration decreasing with increasing porosity. At the lower porosities (below ~60 %), the nanostructure size involved is relatively large (no confinement) and the material can be considered as bulk-like, so as *S* = *S*_d_ + *S*_ph_. Both *S*_d_ and *S*_ph_ increase with decreasing doping concentration in the material. The observed increase in *S* with porosity for porosities up to 60 % can thus be attributed to the decrease in dopant concentration and the increasing internal surface area, which increases carrier scattering.

For porosities above 60 %, the mechanisms involved are more complicated. Si nanowires and nanocrystals become very small and exhibit confinement effects. Their size and percentage in the material change non-monotonically with changing porosity, as demonstrated above from our PL measurements. We have seen that the 63 % porosity sample contained a larger number of light emitting tiny nanocrystals than the 70 % porosity sample. It is thus very difficult to separate the contribution of the different effects involved. The internal surface area is very large, contributing to very strong boundary scattering. There are interesting results in the literature concerning the Seebeck coefficient of Si nanowires and nanocrystals. Boukai et al. [7] studied highly doped individual Si nanowires of diameter 10 and 20 nm and reported an anomalous phonon drag enhancement at room temperature. A recent study on an array of Si nanowires with diameter ~100 nm and doping concentration ~10^19^ cm^−3^ (surface roughness 0.4 nm) [30] contradicts the above and concludes that at 300 K, boundary scattering of phonons in the specific Si nanowires (which were highly doped) completely quenches drag and reduces *S*. If we now consider Si nanocrystals, recent studies [32] demonstrated experimentally that in quite small nanocrystals, of diameter 2.4 nm, the Seebeck coefficient was by one order of magnitude lower than in nanocrystals with sizes of 5.6 and 8.3 nm. It was also reported in the above reference that the 2.4-nm nanocrystals exhibited a threefold stronger coupling of carriers with LO and acoustic phonons; they were thus expected to show higher Seebeck coefficient, which was not the case. This discrepancy was attributed by the authors to strong scattering of phonons and likely reduction of phonon lifetime with decreasing nanocrystal size, resulting in much shorter carrier diffusion and reduced phonon drag, with corresponding reduction in thermopower.

Based on the above discussion, we conclude that the results presented in Fig. 6 can be explained as follows: at low porosities, the increase in *S* with increasing porosity is due to the decreasing carrier concentration, resulting in both an increasing *S*_d_ due to energy filtering effects and an increasing *S*_ph_. At higher porosities, the very strong increase in boundary scattering due to the decrease in nanowire and nanocrystal size, together with the confinement effects involved, results in reduced carrier diffusion and a quenching of phonon drag, with consequent reduction in the total *S*.

## Conclusions

In conclusion, we systematically measured the in-plane Seebeck coefficient of free-standing porous Si membranes with porosities 40–84 % and anisotropic structure, comprising vertical pores and vertical Si nanowires, decorated with nanocrystals. An increase in *S* with increasing porosity is observed, reaching a maximum value of ~1 mV/K at 51 % porosity. Further increase of the porosity leads to a sharp decrease in the Seebeck coefficient, which stabilizes at a value of ~600 μV/K. The initial increase in *S* with porosity is mainly attributed to energy filtering and phonon drag effects. At very high porosities, the material structure is more complicated. Scattering effects become very strong, but also carrier confinement is important, resulting in reduced carrier diffusion and phonon drag quenching.
